# New Miconazole Salts with Heterocyclic Carboxylic Acids with Improved Water Solubility and Enhanced Antifungal Activity

**DOI:** 10.3390/molecules31101686

**Published:** 2026-05-16

**Authors:** Anna Ben, Aleksandra Felczak, Michał Gacki, Katarzyna Lisowska, Mateusz Rafał Gołdyn, Elżbieta Bartoszak-Adamska, Lilianna Chęcińska

**Affiliations:** 1 Faculty of Chemistry, University of Lodz, Pomorska 163/165, 90-236 Lodz, Poland; anna.ben@edu.uni.lodz.pl; 2 Doctoral School of Exact and Natural Sciences, University of Lodz, Narutowicza 68, 90-136 Lodz, Poland; 3 Faculty of Chemistry, Adam Mickiewicz University, Uniwersytetu Poznańskiego 8, 61-614 Poznan, Poland; mateusz.goldyn@amu.edu.pl (M.R.G.); elzbieta.bartoszak-adamska@amu.edu.pl (E.B.-A.); 4 Department of Industrial Microbiology and Biotechnology, Faculty of Biology and Environmental Protection, University of Lodz, Banacha 12/16, 90-237 Lodz, Poland; aleksandra.felczak@biol.uni.lodz.pl (A.F.); katarzyna.lisowska@biol.uni.lodz.pl (K.L.); 5 Institute of General and Ecological Chemistry, Lodz University of Technology, Żeromskiego 114, 90-924 Lodz, Poland; michal.gacki@p.lodz.pl; 6 Center for Advanced Technologies, Adam Mickiewicz University, Uniwersytetu Poznańskiego 10, 61-614 Poznan, Poland

**Keywords:** antifungal activity, haemolysis, heterocycles, miconazole, molecular salt, water solubility

## Abstract

Miconazole is a commonly used imidazole antifungal drug with a broad spectrum of activity against *Candida* strains and other microorganisms. However, its poor solubility and low bioavailability have limited its use to topical infections. To overcome this limitation through the use of cocrystalization techniques, the present work focuses on the relatively less explored class of heterocyclic carboxylic acid coformers, containing two nitrogen atoms in the ring, aimed at developing alternative multicomponent forms of miconazole. Five new forms of miconazole were subjected to in-depth structural analysis, including an evaluation of the effect of hydrate formation. Furthermore, layered motifs in the supramolecular crystal architectures were subjected to qualitative and quantitative surface analysis using CSD-Particle. All new forms of miconazole were also characterized by FT-IR spectroscopy and thermogravimetric analysis. Water solubility was identified as the most important physicochemical property, and significant improvements were obtained for four of the five salts studied. Notably, the newly synthesized miconazole salts with heterocyclic (di)carboxylic acids exhibited high antifungal activity. The tested compounds effectively inhibited the growth of *C. albicans* and *C. parapsilosis* at concentrations several times lower than the parent drug and also showed activity against the important *C. auris* strain. Therefore, the obtained salts may constitute attractive alternatives to currently used antifungal therapies.

## 1. Introduction

Miconazole (Mic) (C_18_H_14_Cl_4_N_2_O, CAS No. 22916-47-8), chemically designated as (*RS*)-1-[2-(2,4-dichlorobenzyloxy)-2-(2,4-dichlorophenyl)ethyl]-1*H*-imidazole, is an antifungal agent active against a broad spectrum of fungi, yeasts, and some Gram-positive bacteria [1,2,3]. Miconazole is classified as a Biopharmaceutics Classification System (BCS [4]) class II compound, characterized by low solubility (less than 1 µg/mL [5] or ~0.4·10^−2^ μg/mL [6]) and high permeability.

The development of multicomponent forms, such as cocrystals, molecular salts, and cocrystals of salts (ionic cocrystals), has become an effective strategy for improving the physicochemical properties of drugs without changing their molecular structure [7,8]. Central to this approach is the concept of supramolecular synthons [9], which guide the design and assembly of multicomponent crystals through predictable intermolecular interactions. The careful selection of suitable coformers, combined with the principles of crystal engineering [10,11], enables the formation of multicomponent crystals with active pharmaceutical ingredients (APIs). This strategy effectively addresses challenges such as poor solubility, limited bioavailability, and reduced stability, which directly affect drug efficacy [12].

Crystallization by slow solvent evaporation is a simple and commonly used technique for obtaining new multicomponent forms of active pharmaceutical ingredients. During crystallization, solvent molecules may be incorporated into the crystal lattice of APIs, leading to the formation of solvates [13]. When water serves as a solvent, the resulting forms are termed hydrates [14,15]. Because water molecules possess both hydrogen-bond donor and acceptor sites, hydrates represent the most common type of multicomponent solid forms [16]. In the pharmaceutical industry, solvates and hydrates play a crucial role, as they often exhibit physicochemical properties, such as solubility, stability, and dissolution rate, distinct from those of their anhydrous counterparts [17]. In some cases, salt hydrates can exhibit lower solubility than anhydrous forms, due to lattice stabilization through extensive hydrogen bonding with water molecules [18]. Consequently, understanding and controlling solvate and hydrate formation is crucial in solid-form screening and drug development.

Several multicomponent cocrystals or salts of miconazole have been reported with small aliphatic dicarboxylic acids such as succinic, fumaric, maleic, tartaric, and adipic acids [5,6,19]. New solid forms of miconazole have also been obtained with trithiocyanuric acid [20], naphthalene-1,5-disulfonic acid [21], and methanesulfonic acid [22]. Drozd et al. demonstrated enhanced solubility of miconazole derivatives compared with their free base and nitrate salt, with the most pronounced improvement observed for the salt with tartaric acid [19], while Tsutsumi et al. reported an improved dissolution rate and superior stability for the hemisuccinate form [5]. Recently, Kanagavel et al. published multicomponent solid forms of miconazole with various coformers, such as sorbic acid, gallic acid, *p*-toluic acid, *p*-anisic acid, 2-chloro-4-nitrobenzoic acid, *α*-lipoic acid, syringic acid, and sinapic acid [23]. These forms have been shown to exhibit improved physicochemical and antifungal properties compared to the parent drug. Several solvatomorphs of miconazole have also been described, namely monohydrate, ethanol monosolvate, methanol monosolvate, and hemihydrate [24,25,26]. Kersten et al. reported that the hemihydrogen peroxide solvate exhibited greater solubility than both the hemihydrate and anhydrous forms of miconazole [27].

This study focuses on the relatively less explored class of heterocyclic carboxylic acid coformers, containing two nitrogen atoms in the ring, aimed at developing alternative multicomponent forms of miconazole. These two nitrogen atoms can occupy different positions in the ring, resulting in three structural isomers: 1,2-diazine (pyridazine), 1,3-diazine (pyrimidine), and 1,4-diazine (pyrazine) [28]. They belong to classes of compounds that possess a wide spectrum of pharmacological activities. Heterocyclic carboxylic acids exhibit a variety of biological properties, including antibacterial, antifungal, anti-inflammatory, and anti-cancer activities [29,30]. Pyrimidines are components of nucleic acids and are of great importance [31]; examples include uracil, which is found in ribonucleic acid; thymine, which is found in deoxyribonucleic acid; and cytosine, which is found in both types of nucleic acid [32,33]. Pyridazine and its derivatives exhibit a wide variety of biological activities, including antibacterial, antifungal, antitubercular, antiviral, anticancer, antihypertensive, diuretic, and anticoagulant properties [34,35].

The objective of this study is to enhance the water solubility of miconazole through the cocrystallization with heterocyclic carboxylic acids and to evaluate its antifungal activity against four *Candida* strains. In this context, novel multicomponent forms of miconazole with coformers, such as pyrazine-2-carboxylic acid (Pyz2CA), pyrazine-2,3-dicarboxylic acid (Pyz2,3DCA), pyridazine-3-carboxylic acid (Pyd3CA), and pyrimidine-5-carboxylic acid (Prm5CA), are reported.

Invasive fungal infections are becoming a growing worldwide concern. It is estimated that *Candida* and *Aspergillus* genera may contribute to as many as 2.5 million deaths annually worldwide. This problem has been poorly understood and studied in recent years, prompting the World Health Organization (WHO) to establish a list of fungal priority pathogens in 2022 [36]. This group includes, among others, strains such as *C. albicans*, *C. auris,* and fluconazole-resistant *C. parapsilosis* [37,38]. *Candida* strains are the most important fungal pathogens in humans and can contribute to the development of various diseases, ranging from infections of the mucous membranes and skin to serious, life-threatening diseases. Invasive candidiasis occurs most often among patients with immune deficiencies, including HIV carriers, organ transplant recipients, untreated diabetics, and patients with other co-infections. It is estimated that invasive candidiasis affects approximately 1.6 million people annually, and its mortality rate exceeds 60% [39]. The treatment of fungal infections is currently limited to only a few drugs belonging to four chemical classes, including, among others, polyenes and azoles. Moreover, in recent years, only a few compounds have met the criteria for antifungal drugs and have been approved for use in fungal infections. These factors, together with the growing problem of drug resistance, have made the search for new effective antifungal drugs a global challenge.

## 2. Results and Discussion

### 2.1. Molecular Features

**Figure 1:**
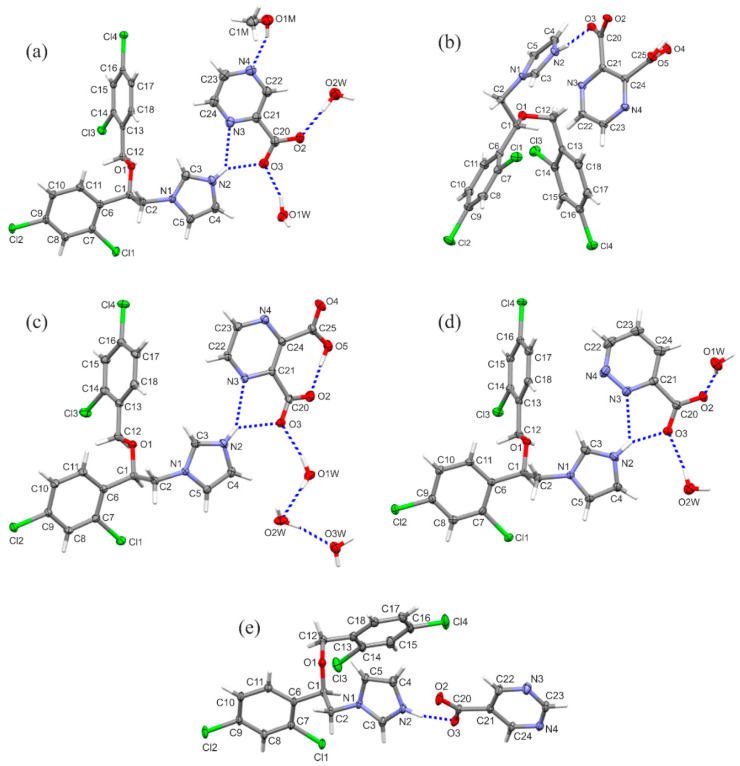
View of the asymmetric unit of (**a**) Mic^+^·Pyz2CA^−^·2H_2_O·0.5MeOH (**1**), (**b**) Mic^+^·Pyz2,3DCA^−^ (**2**), (**c**) Mic^+^·Pyz2,3DCA^−^·3H_2_O (**3**), (**d**) Mic^+^·Pyd3CA^−^·2H_2_O (**4**), and (**e**) Mic^+^·Prm5CA^−^ (**5**), with atom-numbering schemes. In (**1**), the methanol solvent molecule has a half-occupancy. Displacement ellipsoids are drawn at the 50% probability level. H-atoms are shown as spheres of arbitrary radii.

The molecular structures of the asymmetric unit of the miconazole salts (**1**)–(**5**) are presented in Figure 1 with the atom numbering scheme and anisotropic displacement ellipsoids. In all compounds, the miconazole cation is formed by protonation of the nitrogen atom (N2) of the imidazole group, as confirmed by the C3—N2—C4 valence angle, which ranges from 108.7(2)° in (**5**) to 109.4(2)° in (**4**). For comparison, the same angle in the neutral imidazole ring is typically 103–106° [20]. Additionally, the location of the imidazolium hydrogen atom was confirmed from the difference Fourier maps.

In all salt structures considered in this study, miconazole cations adopt three different molecular conformations, as illustrated in Figure S1 in the Supporting Information. These conformations differ in the mutual orientations of the three ring fragments: two phenyl rings and one imidazole ring. The first type of conformation occurs in (**1**), (**3**) and (**4**); the second type in (**2**); and the third in (**5**). Following Drozd et al. (2021), the torsion angle C1—O1—C12—C13 (Table S1 in the Supporting Information), which differentiates the molecular conformations of miconazole, is critical for crystal packing density; a lower packing density of the molecules in a crystal corresponds to a larger torsion angle [6].

**Scheme 1:**
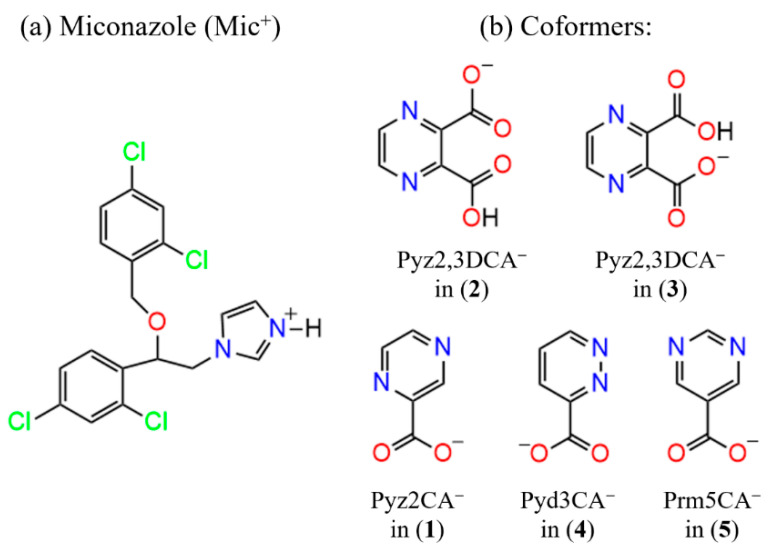
The chemical structures of (**a**) miconazole cation and (**b**) the heterocyclic (di)carboxylic acid coformers used in this work.

In the Pyz2,3DCA and Prm5CA anions, the negative charge of the carboxylate groups is weakly delocalized, as indicated by the C—O bond lengths deviating by more than 3σ, whereas in the Pyz2CA and Pyd3CA anions, the corresponding bond lengths are more comparable (Table S2 in the Supporting Information). The protons of the carboxylic groups in pyrazine-2,3-dicarboxylic acid (Pyz2,3DCA) in the salts (**2**) and (**3**) were clearly localized on the oxygen atom involved in the single C—O bond (~1.3 Å) from the difference Fourier maps. Interestingly, the Pyz2,3DCA anion exhibits distinct molecular conformations, as shown in Scheme 1 and Figure 1b,c. In the hydrate form (**3**), the Pyz2,3DCA anion contains an intramolecular O5—H5A···O2 hydrogen bond, which forms an additional S(7) ring motif [40,41] fused with the heterocyclic ring. As a result, the entire anion molecule is nearly planar in contrast to the solvent-free structure (**2**), where the two carboxylic groups form a dihedral angle of 58.5(1)° and are differently oriented to the heterocyclic ring with dihedral angles of 36.1(1)° and 60.3(1)°, respectively.

### 2.2. Crystal Architectures

The supramolecular characterization of the five salts of miconazole only considers the most structurally significant interactions, those shorter than the sum of the van der Waals radii of the interacting atoms. In (**1**), the methanol molecule disordered about the inversion center was excluded from the analysis; however, it acts as a hydrogen-bond donor to the nitrogen atom of the Pyz2CA anion (O1M···N4 = 2.80(2) Å) and as a hydrogen-bond acceptor from a water molecule (O2*W*···O1M = 2.84(3) Å). The geometrical parameters describing hydrogen bonds in the crystal structures of (**1**)–(**5**) are summarized in Table 1, while aromatic π–π stacking interactions are listed in Table S3 in the Supporting Information. Note that the hydrogen-bond notation used in the structural analysis does not indicate the charges of donors and acceptors.

**Figure 2:**
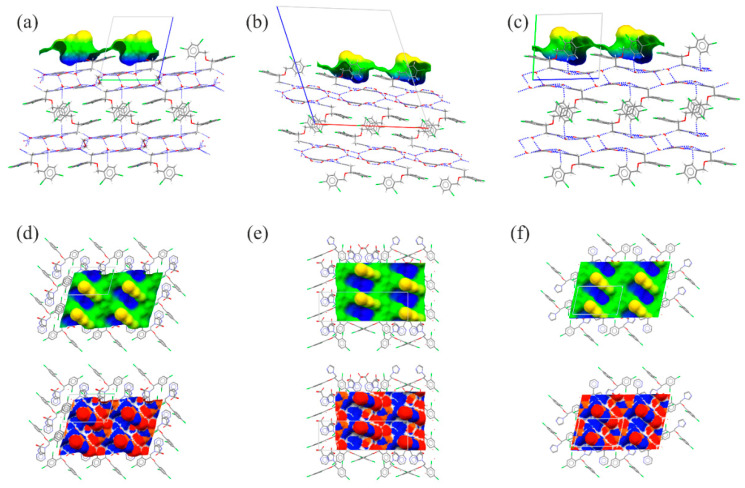
Side-on view of the surface: (**a**) (002) in Mic^+^·Pyz2CA^−^·2H_2_O·0.5MeOH (**1**), (**b**) (002) in Mic^+^·Pyz2,3DCA^−^·3H_2_O (**3**), and (**c**) (020) in Mic^+^·Pyd3CA^−^·2H_2_O (**4**). (**d**–**f**) The corresponding top-down view of the surface topology (green indicates the height around the mean plane with blue below and yellow above the plane) and atom properties: H-bond acceptors (red), H-bond donors (blue), and aromatic (orange).

The molecular salt Mic^+^·Pyz2CA^−^·2H_2_O·0.5MeOH (**1**) crystallizes in the triclinic space group *P*$\bar{1}$. The miconazole cation interacts with the 2-pyrazinecarboxylate anion in a donor-bifurcated manner via N2—H2···O3 and N2—H2···N3 hydrogen bonds. The two water molecules (and a half of the methanol molecule) link these molecular ionic pairs into di-periodic layers parallel to the (001) plane (Figure 2a). The C—H···O contacts and aromatic π–π stacking interactions, *Cg*1···*Cg*4 and *Cg*2···*Cg*4 (where *Cg*1, *Cg*2, and *Cg*4 correspond to the centers of gravity of the imidazole, C6—C11 phenyl, and pyrazine rings, respectively) further stabilize the layer formation.

The C13—C18 phenyl groups of the miconazole cation stick out of the layer and form characteristic aromatic π–π stacks between arene rings (*Cg*3···*Cg*3) of adjacent layers. Between the interpenetrating layers, a halogen bond C14—Cl3···N3(−*x* + 1, −*y* + 1, −*z* + 1) is also observed with a short Cl3···N3 contact distance of 3.169(2) Å and a C14—Cl3···N3 angle of 154.4(1)°.

**Figure 3:**
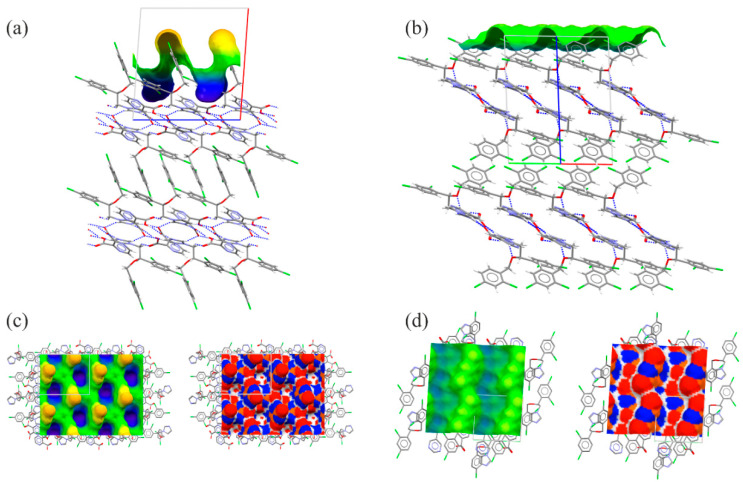
The side-on view of the surface: (**a**) (200) in Mic^+^·Pyz2,3DCA^−^ (**2**) and (**b**) (001) in Mic^+^·Prm5CA^−^ (**5**). (**c**,**d**) The corresponding top-down view of the surface topology (green indicates the height around the mean plane with blue below and yellow above the plane) and atom properties: H-bond acceptors (red), H-bond donors (blue), and aromatic (orange).

The molecular salt Mic^+^·Pyz2,3DCA^−^ (**2**) crystallizes in the monoclinic space group *P*2_1_/c. The monoprotonated acid molecules interact with each other via an O5—H5A···O3 hydrogen bond, forming a characteristic zig-zag chain motif along the crystallographic *b* axis. The miconazole cations are attached to this chain through an N2—H2···O3 hydrogen bond, supported by two additional C4—H4···O2 and C5—H5···N4 contacts. Two further C—H···O interactions, C2—H2*A*···O4 and C3—H3···O2, link adjacent chains into a di-periodic molecular layer parallel to the (100) plane (Figure 3a). The aromatic π–π stacking interaction *Cg*4···*Cg*2 stabilizes the layer formation.

Between adjacent layers, a C15—H15···Cl1(−*x* + 1, *y* − 1/2, −*z* + 3/2) interaction and a single *Cg*3···*Cg*3 contact are observed in contrast to structure (**1**); the latter type of contacts do not form π–π stacks.

The molecular salt Mic^+^·Pyz2,3DCA^−^·3H_2_O (**3**) crystallizes in the monoclinic space group *C*2/c and represents a trihydrated form of structure (**2**). The crystal architecture is significantly influenced by the presence of three water molecules within the crystal lattice. Each acid molecule interacts with three distinct water molecules: specifically, the O1W water molecule connects with one other water molecule, the O3W interacts with two, and the O2W is surrounded by three water molecules. Ultimately, the acid and water molecules assemble into molecular layers parallel to the (001) plane (Figure 2b).

Miconazole cations are attached to the acid molecules through a bifurcated donor hydrogen bond: N2—H2···O3 and N2—H2···N3 and to water molecules via a C3—H3···O1*W* contact. Between layers, as in structure (**1**), aromatic π–π stacks between arene rings (*Cg*3···*Cg*3) are formed. Moreover, a halogen bond C14—Cl3···N3 (−*x* + 1, −*y*, −*z* + 1) is also observed: a Cl3···N3 contact distance of 3.141(1) Å and a C14—Cl3···N3 angle of 156.1(1)°.

Considering the three molecules in the asymmetric unit, and the fact that each water molecule interacts with at least one other water molecule, the crystals of (**3**) can be regarded as ‘aqua complexes’, according to the classification proposed by Wells (1984) [42] and Khankari and Grant (1995) [43].

The molecular salt Mic^+^·Pyd3CA^−^·2H_2_O (**4**) crystallizes in the triclinic space group *P*$\bar{1}$. The miconazole cation interacts with the pyridazine-3-carboxylate anion through a bifurcated donor hydrogen bond: N2—H2···O3 and N2–H2···N3, as observed in structures (**1**) and (**3**). However, in this case, each acid molecule is linked to two distinct water molecules, which do not interact with each other. One water molecule (O2W) connects acid molecules into a chain motif extending along the *a* axis, while the second water molecule (O1W) bridges two centrosymmetrically related chains to form a double-chain motif. C5—H5···O1*W* interactions assemble these chains into molecular layers parallel to the (010) plane (Figure 2c).

Between layers, as in structures (**1**) and (**3**), characteristic aromatic π–π stacks between arene rings (*Cg*3···*Cg*3) are observed. No other specific close contacts are observed between the layers.

The molecular salt Mic^+^·Prm5CA^−^ (**5**) crystallizes in the triclinic space group *P*$\bar{1}$. Within the asymmetric unit, ionized molecules are connected through an N2—H2···O3 hydrogen bond. Two such ionic pairs are further centrosymmetrically linked via the C5–H5···O2 and C22—H22···O1 interactions, generating three second-level hydrogen-bonded edge-fused rings: R(11)22, R(16)44 and R(24)44. An additional C24—H24···O3 interaction duplicates the basic ionic pair, forming another ring motif R(10)22. Together, these interactions produce a mono-periodic chain-of-rings motif extending along the [110] direction (Figure 3b). This packing arrangement facilitates aromatic π–π interactions between the imidazole and pyrimidine rings, although the dihedral angle between their planes increases to 21.98(13)°. From another side, adjacent chains also interact through a single Cg3···Cg3 contact, similar to structure (**2**); however, the orientations of the outer arene rings differ substantially. Additionally, a halogen bond C7—Cl1···Cl2(*x* + 1, *y*, *z*) is also observed with a short Cl1···Cl2 contact distance of 3.2396(8) Å and a C7—Cl1···Cl2 angle of 129.56(9)°.

### 2.3. Hydrate Formation

In this study, three of the five crystal structures are hydrates with an average number of water molecules per structure greater than one, thus showing the importance of di- or tri-hydrates for salts of miconazole with heterocyclic (di)carboxylic acids containing more than one nitrogen atom in the ring. For comparison, three of four miconazole salts with isomeric pyridine-2,*n*-dicarboxylic acids (*n* = 3, 4, 5, 6) are monohydrates [44]. Thus, a tendency of miconazole salts with heterocyclic coformers to form hydrates is observed. The presence of water molecules affects the level of intermolecular interactions (internal energy and enthalpy) and the degree of crystalline disorder (entropy); hence, it impacts the thermodynamic parameters, solubility, and stability of hydrated active pharmaceutical ingredients (APIs) as well as their mechanical properties [16,43]. As a result, the physical properties of hydrates differ from those of the anhydrous form.

In our study, pyrazine-2,3-dicarboxylic acid forms both anhydrous (**2**) and trihydrate (**3**) salts with miconazole, and interestingly, Mic^+^·Pyz2,3DCA^−^·3H_2_O (**3**) is the only example of an ‘aqua complex’ among the three hydrates considered here. It probably has an impact on water solubility, which is discussed in further detail. In the two remaining hydrated structures, water molecules do not interact with each other; however, despite the different synthons formed by water molecules, similar layered supramolecular substructures in (**1**), (**3**), and (**4**) can be distinguished, which were analyzed using surface-particle analysis.

Moreover, in the three hydrated structures of (**1**), (**3**), and (**4**), the donor:acceptor ratios (D/A) are higher (6:7; 8:8; 5:6, respectively) compared to the ratios observed for the anhydrous structures, namely D/A = 2:5 for (**2**) and D/A = 1:4 for (**5**) (Table S4 in the Supporting Information). This is in line with Desiraju’s findings for hydrated structures [45], which indicate that the number of hydrogen-bond donors is generally much lower than the number of acceptors. However, according to the observations from the CSD reported by van de Streek and Motherwell, unsatisfied donors appear to be the main driving force for water incorporation, since unsatisfied hydrogen-bond donors are highly unfavorable for crystal structures, whereas crystal structures can tolerate relatively high levels of unsatisfied hydrogen-bond acceptors [46].

### 2.4. Surface Analysis with CSD-Particle

As discussed above, the analyzed crystal architectures (**1**)–(**5**) exhibit layered supramolecular substructures. By investigating these layers more deeply through the major particle surfaces of crystalline materials, it is possible to gain a better understanding of how they might interact with other particles and surfaces. To investigate, both qualitatively and quantitatively, the selected surfaces related to the supramolecular planes, the surface analysis feature in CSD-Particle was employed. The surfaces under investigation are the (002) in (**1**), the (200) in (**2**), (002) in (**3**), (020) in (**4**), and (001) in (**5**), respectively, and Table 2 contains the chemical surface descriptors for them. Figure 2 shows that the topology of the three representative surfaces for (**1**), (**3**), and (**4**) is highly similar. These structures possess the same conformation of miconazole cation, are all hydrates, and the ionic molecular pairs from the asymmetric unit are formed via the bifurcated donor hydrogen bond in the same manner. As described, the miconazole cations protrude from the layer with the dichlorophenyl group C13—C18 (attached to the O1 oxygen atom) exposed from the surface. This results in comparable surfaces that are moderately rough, which is confirmed by the numerical values of rugosity, root mean square deviation (RMSD), skewness, and kurtosis (Table 2), and the contour maps show that peaks (yellow) and valleys (blue) are positioned directly adjacent to one another. As shown in Table 3, the densities of the aromatic bonds and H-bond donors are very similar.

The (200) surface in Mic^+^·Pyz2,3DCA^−^ (**2**) and the (001) surface in Mic^+^·Prm5CA^−^ (**5**) exhibit a different topology from that described for structures (**1**), (**3**), and (**4**), which is related to a different conformation of the miconazole cation and the anhydrous forms (Figure 3). The (001) surface in (**5**) is the least rough among all analyzed surfaces, in contrast to the (200) surface in (**2**), for which the roughness is the highest. Interestingly, the surface in (**2**) has peaks and valleys of comparable height, as described by a skewness of 0.096, compared to 0.168 for (**5**); a value of 0 corresponds to equal peak and valley heights. In both cases, the analyzed surfaces are dominated by aromatic interactions, which determine their growth direction.

### 2.5. Spectroscopic Analysis

Fourier transform infrared (FT-IR) spectra for all five miconazole salts are shown in Figure S2 in the Supporting Information. The FT-IR spectrum of miconazole (Figure S3) reveals characteristic absorption bands at 817 cm^−1^ (C−Cl band), 1092 cm^−1^ (stretching vibration peak of the C—O—C bond), 1470 cm^−1^ (C−H stretching vibration bending of the two dichlorobenzene groups), and 1589 cm^−1^ (C—C stretching vibration of the two dichlorobenzene groups) [47,48].

The FT-IR spectra for heterocyclic carboxylic acids (Pyz2CA, Pyz2,3DCA, Pyd3CA, Prm5CA; Figure S3 in the Supporting Information) show characteristic bands in the range of 1701 cm^−1^ to 1711 cm^−1^ assigned to C=O stretching vibration. The absorption bands appearing in the range of 1194 cm^−1^ to 1274 cm^−1^ are related to C—N. Around 3400 cm^−1^ characteristic peaks are observed due to the O—H stretching vibration in the acid molecules.

Evidence for salt formation is found in the disappearance of the band associated with the carboxylic acid group; instead, two bands corresponding to deprotonated carboxylate groups appear at 1631 cm^−1^ (asymmetric stretching of COO^−^) and 1350 cm^−1^ (symmetric stretching of COO^−^). Interestingly, in the salt with Pyz2,3DCA^−^, both the 1701 cm^−1^ (C=O) band and the 1631/1350 cm^−1^ carboxylate bands are present simultaneously. This indicates that only one of the carboxylic acid groups is deprotonated. The broad bands observed at 3408 cm^−1^ and 3416 cm^−1^ in (**1**), (**3**), and (**4**) are attributed to the stretching of the O—H bonds of the water molecule.

### 2.6. Thermal Analysis

**Figure 4:**
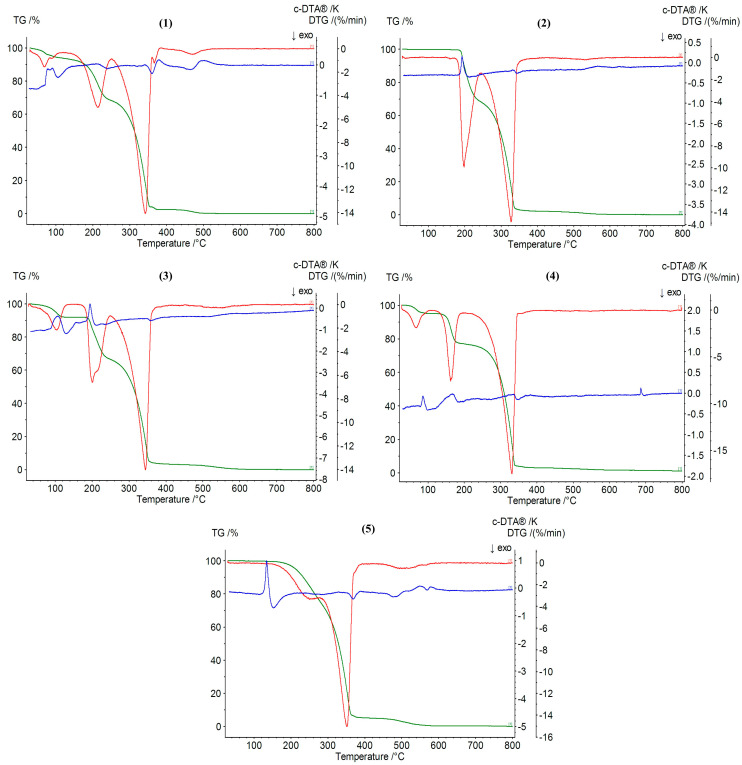
The thermal decomposition of miconazole salts: Mic^+^·Pyz2CA^−^·2H_2_O·0.5MeOH (**1**), Mic^+^·Pyz2,3DCA^−^ (**2**), Mic^+^·Pyz2,3DCA^−^·3H_2_O (**3**), Mic^+^·Pyd3CA^−^·2H_2_O (**4**), and Mic^+^·Prm5CA^−^ (**5**). TG is plotted in green, DTG in red, and DTA in blue.

Thermograms of the miconazole salts (**1**)–(**5**) are shown in Figure 4. Experimental data indicate that compounds (**1**), (**3**), and (**4**) exist as hydrates, whereas (**2**) and (**5**) are anhydrous. The first decomposition step of hydrates (**3**) and (**4**) is associated with a single-stage dehydration process occurring within the temperature ranges of 50–150 °C for (**3**) and 45–100 °C for (**4**). The observed mass losses are as follows: exp. 8.07%, calc. 8.47% for (**3**) and exp. 5.16%, calc. 6.25% for (**4**). In (**1**), the desolvation of a methanol and water molecule proceeds at a temperature range of 38–140 °C (mass loss exp. 7.39% and calc. 8.78%). For (**1**), (**3**), and (**4**), the DTA curves exhibit characteristic endothermic effects corresponding to this process. The second decomposition step of (**1**), (**3**), and (**4**) is attributed to the degradation of relevant organic molecules. The latter are represented by exothermic effects on the DTA curves. Complete decomposition occurs at 530 °C, 630 °C, and 680 °C for (**1**), (**3**), and (**4**), respectively.

Thermal decomposition of the anhydrous compounds (**2**) and (**5**) begins at 192 °C and 140 °C, respectively. These processes precede the endothermic effects related to the melting point on DTA curves. Subsequent heating leads to either two-stage or one-stage degradation of the organic components for (**2**) and (**5**), respectively. Complete decomposition is achieved at 625 °C for (**2**) and 600 °C for (**5**).

The TG curves of all miconazole salts, along with the associated mass losses, suggest that coformers are released first, followed by the miconazole molecules, except for compound (**5**), for which the release sequence could not be determined.

### 2.7. Water Solubility Studies

Miconazole is a weak base with a *p*K_a_ of 6.7 [49]. The free base form is practically insoluble in water with reported solubility values below 1 µg/mL [5] or ~0.4·10^−2^ μg/mL [6]. In the present study, this value could not be experimentally verified due to instrument sensitivity limitations.

HPLC studies have shown an improvement in the solubility of miconazole by using heterocyclic carboxylic acid coformers containing two nitrogen atoms in the ring. The coformers differ in the position of both nitrogen atoms in the ring, as well as the number and position of the carboxyl groups relative to the aromatic nitrogen atoms. This has a clear impact on the determined solubility values of the multicomponent ionic miconazole systems tested. The measured values, expressed as the miconazole concentration in mg/L or mmol/L, are summarized in Table 4.

Among all the systems studied, the greatest improvement in solubility was observed for its salt with Pyd3CA as a coformer. Miconazole in the form of the Mic^+^·Pyd3CA^−^·2H_2_O (**4**) salt hydrate is over 37-fold more soluble than the pure API. Among the obtained salt solvates, Mic^+^·Pyz2,3DCA^−^·3H_2_O (**3**) proved to be the least soluble, with only a 2.5-fold improvement in solubility. The use of the Pyz2,3DCA coformer allowed the preparation and identification of both the anhydrous salt Mic^+^·Pyz2,3DCA^−^ (**2**) and the salt hydrate Mic^+^·Pyz2,3DCA^−^·3H_2_O (**3**). Of these, the anhydrous form (**2**) proved to be more soluble, and analysis showed an over 30-fold improvement in miconazole solubility. A similar trend was observed for multicomponent forms of norfloxacin [18] and theobromine [50]. The solubility of both anhydrous ionic complexes of miconazole (**2**) and (**5**) is comparable. The use of Prm5CA as a coformer allowed the preparation of the Mic^+^·Prm5CA^−^ salt (**5**), which is more than 33 times more soluble than miconazole.

Recently, multicomponent forms of miconazole with the following acids: sorbic acid, gallic acid, *p*-toluic acid, *p*-anisic acid, 2-chloro-4-nitrobenzoic acid, *α*-lipoic acid, syringic acid, and sinapic acid were reported by Kanagavel et al. [23]. In water at 37 °C, all these multicomponent forms of miconazole showed improved water solubility compared to the parent drug, with the exception of miconazole-2-chloro-4-nitrobenzoic acid. Among these cocrystals, miconazole-sorbic acid and miconazole-*p*-toluic acid showed significantly higher water solubility than the others.

### 2.8. Antifungal Activity

Although miconazole is a widely used imidazole antifungal drug with broad activity against *Candida* species, its poor solubility and low bioavailability limit its use to topical applications. To address this limitation using cocrystalization techniques, the present work evaluates the antifungal activity of newly synthesized miconazole salts with heterocyclic (di)carboxylic acids against critically important *Candida* strains causing a serious threat to public health. As shown in Table 4, the obtained miconazole salts are characterized by improved solubility, which affects the antifungal activity of the tested compounds (Table 5). The analyzed salts showed the highest antifungal activity against *C. albicans*. For this strain, the MIC values were 16-fold lower for compound (**5**) and 8-fold lower for compounds (**1**) and (**4**) compared to the MIC value of miconazole. In the case of *C. parapsilosis*, growth inhibition was observed at 2- or 4-fold lower concentrations of tested salts in comparison to miconazole. Importantly, the newly synthesized derivatives also limit the growth of the *C. auris* strain. Compound (**1**) was effective at an 8-fold lower concentration than the standardly used miconazole, while compounds (**2**) and (**5**) showed activity at 2-fold lower concentrations. In the case of *C. glabrata*, the tested compounds showed antifungal properties comparable to those of miconazole. It is worth noting that antifungal activity testing was also conducted for all ligands used in the synthesis. Based on the obtained results, none of the ligands alone inhibited the growth of the tested strains.

The literature data indicate that crystal engineering often yields new multicomponent forms with desirable antifungal activity comparable to that of the parent drug, while substantial improvements in biological efficacy can be difficult to achieve. Martin et al. (2025) [51] obtained cocrystals of ketoconazole-adipic acid with seven pharmaceutical excipients. The reported results indicated that ketoconazole-adipic acid adducts were characterized by improved stability and solubility. Moreover, a molecular docking study demonstrated that the tested cocrystals exhibited higher affinity for sterol 14α-demethylase (CYP51), an enzyme present in yeast *C. albicans*, resulting in improved antifungal properties.

The presented data are consistent with the work of Kanagavel et al. [23], describing the biological properties of multicomponent solid forms of miconazole. The newly synthesized compounds effectively inhibited the growth of the *Candida* strains, with MIC values ranging from 15.6 to 31.2 mg/L. Considering that the experimental MIC values for miconazole were 31.5 mg/L and 15.6 mg/L for *C. albicans* and the other tested *Candida* strains, respectively, it could be concluded that the reported miconazole salts and cocrystals exhibit comparable or even better antifungal activity.

Another study reported that new salts of miconazole and ketoconazole with naphthalene disulfonic acid were characterized by higher stability and solubility than the parent drugs [21]. Biological evaluation of these crystals against *Candida* strains showed that the ketoconazole derivative had better antifungal activity than the corresponding miconazole compound. The MIC90 values for ketoconazole-naphthalene disulfonic acid salts against *C. auris*, *C. parapsilosis,* and *C. glabrata* strains were identical to those presented for the parent drug. In contrast, for miconazole salt to achieve growth inhibition comparable to that of the parent compound, it was necessary to use concentrations 2- or 4-fold higher, depending on the *Candida* strain tested.

### 2.9. Toxicity Studies

Due to the fact that the biological characterization of newly synthesized compounds usually includes the determination of cytotoxicity, the next stage of the work involved a haemolysis assay. This test allows us to determine whether the tested substances interact with the erythrocyte membrane and lead to the release of cellular components. The haemolysis assay is one of the most frequently chosen tests at the initial stage due to its simplicity and repeatability (Sæbø et al., 2023) [52]. The analyzed compounds are considered biocompatible when the level of haemolysis does not exceed 10% (Fabijańska et al., 2024) [53].

**Figure 5:**
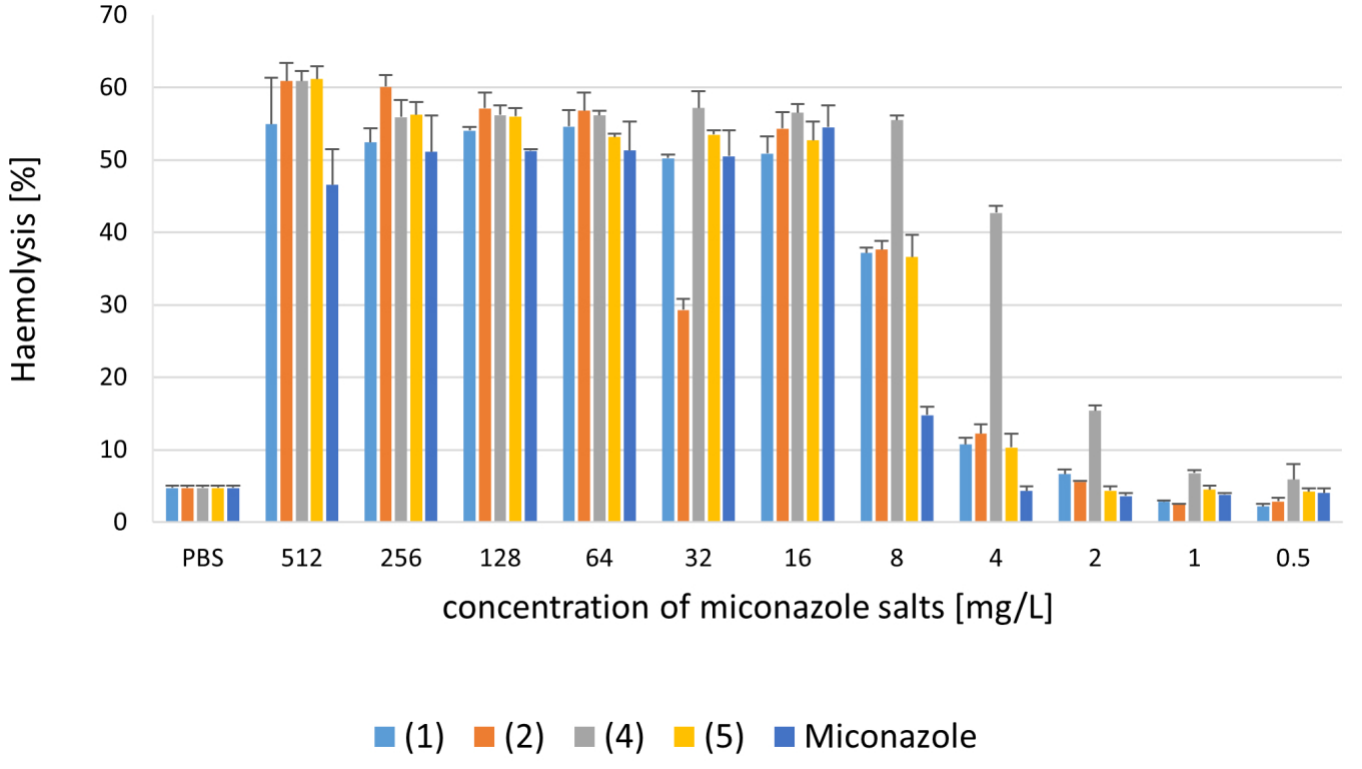
The haemolytic activity of miconazole salts Mic^+^·Pyz2CA^−^·2H_2_O·0.5MeOH (**1**), Mic^+^·Pyz2,3DCA^−^ (**2**), Mic^+^·Pyd3CA^−^·2H_2_O (**4**), and Mic^+^·Prm5CA^−^ (**5**) in comparison to the parent drug.

As shown in Figure 5, the toxicity of the newly synthesized compounds is dose-dependent. In systems containing more than 8 mg/L of the tested compounds, the level of haemolysis is comparable to that observed in the system containing miconazole. In samples containing less than 4 mg/L of miconazole salts, haemolysis drops below 10%, with the exception of compound (**4**). It is worth noting that compound (**1**) can be considered the most biocompatible, because it effectively inhibits the growth of all tested *Candida* strains at concentrations that are not toxic to erythrocytes.

There is limited information in the literature regarding the toxicity of derivatives based on the miconazole structure. Studies by Kanagavel et al. (2026) have shown that miconazole multicomponent solids demonstrate lower cytotoxicity than the parent drug [23]. Additionally, in a study by Fabijańska et al., it was confirmed that miconazole complexes of silver(I) may be less cytotoxic to fibroblasts than miconazole itself.

## 3. Materials and Methods

### 3.1. Materials

All reagents are commercially available and were used without further purification. Miconazole (Mic), pyridazine-3-carboxylic acid (Pyd3CA), and pyrimidine-5-carboxylic acid (Prm5CA) were purchased from Fluorochem (Hadfield, United Kingdom), while pyrazine-2-carboxylic acid (Pyz2CA) and pyrazine-2,3-dicarboxylic acid (Pyz2,3DCA) were purchased from BLD Pharmatech GmbH (Reinbek, Germany). Ethanol from Chempur (Piekary Śląskie, Poland) was analytical grade and was used as received.

### 3.2. Preparation of Miconazole Salts

Equimolar quantities (0.05 mmol of each component) of miconazole and selected coformers (Pyz2CA, Pyz2,3DCA, Pyd3CA, and Prm5CA) were ground together in a mortar and pestle. The resulting fine powder mixtures were dissolved in ethanol (2 mL). The mixtures were heated and stirred until the substances were completely dissolved. The crystals were obtained by slow evaporation from the filtrates under ambient conditions. Melting points: 94 °C (**1**), 195 °C (**2**), 193 °C (**3**), 99 °C (**4**), and 133 °C (**5**).

### 3.3. X-Ray Single-Crystal Diffraction

The crystal data for Mic^+^·Pyz2CA^−^·2H_2_O·0.5MeOH (**1**) was collected using a Bruker D8 QUEST diffractometer equipped with a PHOTON-III detector. The Bruker APEX5 software [54] was used for data collection, integration, reduction, scaling, and refinement of the single-crystal data, and SADABS [55] was used to correct intensities for absorption. The single crystal X-ray diffraction data of Mic^+^·Pyz2,3DCA^−^ (**2**), Mic^+^·Pyz2,3DCA^−^·3H_2_O (**3**), Mic^+^·Pyd3CA^−^·2H_2_O (**4**), and Mic^+^·Pyd3CA^−^ (**5**) were collected at 100 K on a Rigaku XtaLAB Synergy diffractometer equipped with a HyPix or Pilatus 300 K detector. A numerical absorption correction based on the Gaussian integration over a multifaceted crystal model was applied using CrysAlis^Pro^ (Rigaku Oxford Diffraction, Yarnton, Oxfordshire, UK). The X-ray crystal structures were solved by dual-space methods using SHELXT [56] and refined by full-matrix least-squares procedures using SHELXL-2019/3 [57].

The H atoms bonded to C atoms were included in the refinement using riding models, with constrained distances set to 0.95 Å (aromatic), 0.98 Å (methyl), 0.99 Å (methylene), and 1.0 Å (methine), and with *U*_iso_(H) = 1.2*U*_eq_ of the attached C atom.

The hydrogen atoms bonded to heteroatoms (N, O), involved in hydrogen bonds, were located in difference Fourier maps and refined isotropically.

In Mic^+^·Pyz2CA^−^·2H_2_O·0.5MeOH (**1**), the methanol molecule was found to be disordered across the inversion center and modeled at half occupancy; the (O)–H was refined as a riding model [0.84Å, *U*_iso_(H) = 1.5*U*_eq_(O)].

Molecular diagrams and other figures of crystal structures were prepared using Mercury [58].

Crystal data, data collection, and structure refinement details for compounds (**1**)–(**5**) are summarized in Table S5 in the Supporting Information.

Crystal data for (**1**) (*M* = 1184.53 g/mol): triclinic, space group *P*$\bar{1}$ (no. 2), *a* = 8.056(2) Å, *b* = 12.4575(3) Å, *c* = 13.494(3) Å, α = 99.535(1)°, β = 101.902(1)°, γ = 102.819(1)°, *V* = 1259.97(5) Å^3^, *Z* = 2, *T* = 100 K, μ(Cu*K*α) = 4.675 mm^−1^, *D*_calc_ = 1.561 g/cm^3^, 45797 reflections measured (3.4° ≤ Θ ≤ 68.4°), 4612 unique (*R*_int_ = 0.032) which were used in all calculations. The final *R*1 was 0.034 [*I* > 2σ(*I*)], and *wR*2 was 0.092 (all data).

Crystal data for (**2**) (*M* = 584.22 g/mol): triclinic, space group *P*2_1_/c (no. 14), *a* = 15.0239(1) Å, *b* = 11.2852(1) Å, *c* = 14.4304(1) Å, β = 94.141(1)°, *V* = 2440.25(3) Å^3^, *Z* = 4, *T* = 100 K, μ(Cu*K*α) = 4.810 mm^−1^, *D*_calc_ = 1.590 g/cm^3^, 23,877 reflections measured (2.9° ≤ Θ ≤ 77.2°), 4916 unique (*R*_int_ = 0.025) which were used in all calculations. The final *R*1 was 0.027 [*I* > 2σ(*I*)], and *wR*2 was 0.069 (all data).

Crystal data for (**3**) (*M* = 638.27 g/mol): triclinic, space group *C*2/*c* (no. 15), *a* = 25.8049(4) Å, *b* = 7.9664(1) Å, *c* = 28.1278(4) Å, β = 110.143(2)°, *V* = 5428.63(15) Å^3^, *Z* = 8, *T* = 100 K, μ(Cu*K*α) = 4.459 mm^−1^, *D*_calc_ = 1.562 g/cm^3^, 31923 reflections measured (3.3° ≤ Θ ≤ 78.7°), 5678 unique (*R*_int_ = 0.033) which were used in all calculations. The final *R*1 was 0.028 [*I* > 2σ(*I*)], and *wR*2 was 0.072 (all data).

Crystal data for (**4**) (*M* = 576.24 g/mol): triclinic, space group *P*$\bar{1}$ (no. 2), *a* = 8.1485(1) Å, *b* = 12.3256(3) Å, *c* = 12.7127(2) Å, α = 84.538(2)°, β = 77.624(1)°, γ = 84.508(2)°, *V* = 1237.74(4) Å^3^, *Z* = 2, *T* = 100 K, μ(Cu*K*α) = 4.727 mm^−1^, *D*_calc_ = 1.546 g/cm^3^, 23,067 reflections measured (3.6° ≤ Θ ≤ 72.1°), 4756 unique (*R*_int_ = 0.030) which were used in all calculations. The final *R*1 was 0.039 [*I* > 2σ(*I*)], and *wR*2 was 0.100 (all data).

Crystal data for (**5**) (*M* = 540.21 g/mol): triclinic, space group *P*$\bar{1}$ (no. 2), *a* = 8.4747(2) Å, *b* = 8.9484(3) Å, *c* = 15.3128(5) Å, α = 90.364(3)°, β = 94.076(2)°, γ = 91.372(2)°, *V* = 1157.94(6) Å^3^, *Z* = 2, *T* = 100 K, μ(Cu*K*α) = 4.947 mm^−1^, *D*_calc_ = 1.549 g/cm^3^, 12,339 reflections measured (2.9° ≤ Θ ≤ 72.1°), 4407 unique (*R*_int_ = 0.033) which were used in all calculations. The final *R*1 was 0.044 [*I* > 2σ(*I*)], and *wR*2 was 0.127 (all data).

### 3.4. FT-IR Spectroscopy

The Fourier transform infrared (FT-IR) spectra of pure miconazole and five miconazole salts (**1**)–(**5**) were collected using a ThermoScience Nicolet iS5 (Thermo Fisher Scientific, Waltham, MA, USA) in the range of wave numbers from 4000 cm^−1^ to 400 cm^−1^ in the solid state with KBr pellets. Data were analyzed using OMNIC software.

### 3.5. Thermogravimetric Analysis (TGA)

Thermogravimetric analyses (TG/DTG/DTA) were conducted with the Netzsch TG 209F1 Iris thermoanalyzer (Netzsch, Selb, Germany). Compounds (**1**)–(**5**) were heated (in ceramic crucibles) from 27 °C to 800 °C at a heating rate of 10 °C min^−1^ in atmospheric air. The experimental data were processed using the NETZSCH Proteus software (ver. 6.1.0).

### 3.6. Water Solubility Measurements

The solubility of the miconazole base and miconazole in the composition of its salts was measured in triplicate by the shake-flask method at 25 °C [59]. An excess amount of each sample was suspended in 30 mL of distilled water as dissolution medium for 24 h. After that, the suspension was filtered through a microdisc membrane filter with a 0.45 µm pore size (BioSens). The concentration of miconazole in solution was determined using the high-performance liquid chromatography (HPLC) method. The HPLC analysis was performed on a Merck-Hitachi D-7000 HPLC equipped with a UV detector (Hitachi, Tokyo, Japan) and ACE 5 C18 (15 cm × 4.6 mm) column. Elution was achieved by a mobile phase consisting of 0.02% triethylamine and methanol (15:85, *v*/*v*) at a flow rate of 0.9 mL/min [60]. The UV detection of miconazole was carried out using a UV detector set at 225 nm.

### 3.7. Assessment of Antifungal Properties

The antifungal activity of the miconazole salts (**1**), (**2**), (**4**), and (**5**) was determined against four *Candida* strains (*Candida albicans* ATCC 10231, *Candida parapsilosis* ATCC 22019, *Candida glabrata* ATCC 2001, and *Candida auris* CDC B11903). Mic^+^·Pyz2,3DCA^−^·3H_2_O (**3**) was not included in the biological tests because it exhibited the lowest improvement in water solubility among the tested compounds. The analyses were performed using the microdilution method in accordance with the CLSI standard M27 (Fourth Edition) guidelines. The results were expressed as the minimum inhibitory concentration (MIC) and the minimum fungicidal concentration (MFC), where MIC is defined as the lowest concentration of a salt that prevents visible growth of yeast, and MFC is defined as the lowest salt concentration that totally limits the viability of the cells.

### 3.8. Determination of Haemolytic Activity

The red blood cells necessary to assess the toxicity of the studied miconazole salts were obtained from the Regional Center of Blood Donation and Blood Treatment in Lodz (Poland). Erythrocytes were washed four times with PBS and then diluted in solutions of tested miconazole salts to achieve a 2% haematocrit. At the same time, a negative control (containing erythrocytes and PBS) and a positive control (red blood cells and a solution of Triton X-100) were prepared. Next, the samples were incubated for 24 h at 37 °C in the dark. After the indicated time, the probes were centrifuged for 15 min at 3000 rpm. The amount of released haemoglobin from erythrocytes was measured in the obtained supernatants at λ = 540 nm using a MultiskanTM FC Microplate Photometer (Thermo Fisher Scientific, Pudong, Shanghai, China). The haemolytic activity of the studied miconazole salts was calculated using the following formula:

% Haemolysis = *As*/*Ac* × 100%

where *As* is the absorbance of the sample and *Ac* is the absorbance of the sample containing erythrocytes and Triton X-100 (100% of haemolysis).

## 4. Conclusions

Crystal engineering of multicomponent solid forms represents a rational approach to modulating the molecular organization in the solid state, improving water solubility and, consequently, enhancing antimicrobial activity. The present study fits well within this framework. Five new organic salts were obtained by cocrystallizing miconazole with heterocyclic carboxylic acids. Carboxylic acids of isomeric diazines were used as coformers, including pyrazine-2-carboxylic acid, pyrazine-2,3-dicarboxylic acid, pyridazine-3-carboxylic acid, and pyrimidine-5-carboxylic acid. The presence of two nitrogen atoms in the aromatic ring supports the formation of hydrogen bonds between the drug molecule and the coformer. The most frequently observed supramolecular motif is a layered structure, whose formation is influenced by both the molecular conformation of miconazole and the presence of water molecules. Our observations confirm that water molecules pose a challenge in the context of crystal engineering [61].

Among the obtained salts, two structures can be distinguished with the same coformer pyrazine-2,3-dicarboxylic acid (Pyz2,3DCA): one anhydrous and one trihydrate. The thermal behavior of the anhydrous salt (**2**) and the hydrated salt (**3**) differs solely due to the presence of water molecules in the crystal lattice. After dehydration, both compounds exhibit identical thermal behavior, including the same endothermic effect associated with melting, two decomposition stages, and comparable temperatures for complete decomposition.

The effect of hydration on the solubility of these salts is significant. The trihydrate is the only compound among those studied that exhibits relatively low solubility in water. This behavior is likely related to the aqua complex structure, in which water molecules interconnect in a chain pattern that permeates the crystal layer architecture.

The cytotoxicity of the obtained salts was determined using a hemolysis assay, and their antifungal activity was also evaluated. Most importantly, the salts effectively inhibited the growth of *C. albicans* and *C. parapsilosis* at concentrations significantly lower than that of the parent miconazole drug. Moreover, they also exhibited high antifungal activity against *C. auris* and *C. glabrata*. Therefore, the obtained salts not only demonstrate the potential of crystal engineering for tuning solid-state properties but also represent promising candidates for the development of more effective therapies with improved solubility and bioavailability.

## Figures and Tables

**Table 1:** Hydrogen-bond geometry for (**1**)–(**5**).

D—H···A	D—H [Å]	H···A [Å]	D···A [Å]	∠D—H···A [°]
Mic^+^·Pyz2CA^−^·2H_2_O·0.5MeOH (**1**)				
N2—H2···O3	0.89 (3)	1.93 (3)	2.709 (2)	145 (2)
N2—H2···N3	0.89 (3)	2.38 (3)	3.099 (3)	138 (2)
C2—H2*B*···O2*W*^i^	0.99	2.49	3.382 (3)	150
C3—H3···O1*W*^ii^	0.95	2.34	3.228 (3)	156
C4—H4···O1*W*	0.95	2.52	3.363 (3)	148
C18—H18···O1*W*^ii^	0.95	2.50	3.410 (3)	161
C23—H23···O2^ii^	0.95	2.51	3.310 (3)	142
O1*W*—H1*W*1···O2^iii^	0.96 (2)	1.90 (2)	2.818 (2)	159 (2)
O1*W*—H2*W*1···O3	0.97 (2)	1.87 (2)	2.790 (2)	158 (3)
O2*W*—H1*W*2···O2	0.98 (2)	1.82 (2)	2.794 (3)	173 (3)
Mic^+^·Pyz2,3DCA^−^ (**2**)				
N2—H2···O3	0.88 (2)	1.83 (2)	2.7030 (15)	168 (2)
O5—H5*A*···O3^i^	0.92 (2)	1.65 (2)	2.5456 (13)	166 (2)
C2—H2*A*···O4^ii^	0.99	2.38	3.2372 (16)	145
C3—H3···O2^iii^	0.95	2.28	3.1927 (16)	162
C4—H4···O2^iv^	0.95	2.23	3.1658 (16)	168
C5—H5···N4^v^	0.95	2.58	3.5230 (17)	170
C15—H15···Cl1^vi^	0.95	2.76	3.6364 (14)	153
Mic^+^·Pyz2,3DCA^−^·3H_2_O (**3**)				
N2—H2···O3	0.90 (2)	2.06 (2)	2.7574 (16)	133 (2)
N2—H2···N3	0.90 (2)	2.28 (2)	3.1010 (17)	152 (2)
O5—H5*A*···O2	0.89 (2)	1.52 (2)	2.4055 (16)	173 (3)
C3—H3···O1*W*^i^	0.95	2.10	3.0274 (18)	164
O1*W*—H1*W*1···O3	0.88 (3)	1.88 (3)	2.7522 (15)	170 (2)
O1*W*—H2*W*1···O2*W*	0.84 (2)	2.11 (3)	2.9395 (18)	171 (2)
O2*W*—H1*W*2···O3*W*	0.83 (2)	1.92 (2)	2.7327 (19)	164.1 (19)
O2*W*—H2*W*2···O4^ii^	0.81 (3)	2.20 (3)	2.9643 (17)	158 (2)
O2*W*—H2*W*2···O5^ii^	0.81 (3)	2.52 (3)	3.1858 (17)	141 (2)
O3*W*—H1*W*3···O4^iii^	0.86 (3)	2.47 (3)	3.0830 (17)	128 (2)
O3*W*—H1*W*3···N4^iii^	0.86 (3)	2.11 (3)	2.9376 (18)	161 (3)
O3*W*—H2*W*3···O2*W*^iv^	0.807 (19)	2.09 (2)	2.876 (2)	165.5 (18)
Mic^+^·Pyd3CA^−^·2H_2_O (**4**)				
N2—H2···O3	0.95 (4)	1.80 (4)	2.686 (3)	154 (4)
N2—H2···N3	0.95 (4)	2.39 (4)	3.066 (3)	128 (3)
C4—H4···O2*W*	0.95	2.48	3.254 (4)	139
C5—H5···O1*W*^i^	0.95	2.49	3.240 (4)	136
C18—H18···O2*W*^ii^	0.95	2.52	3.391 (4)	153
C24—H24···O2^ii^	0.95	2.35	3.301 (4)	175
O1*W*—H1*W*1···O2^iii^	0.83 (2)	2.06 (2)	2.864 (3)	164 (4)
O1*W*—H2*W*1···O2	0.85 (2)	1.99 (2)	2.811 (3)	162 (4)
O2*W*—H1*W*2···O3	0.85 (2)	1.94 (2)	2.783 (3)	170 (5)
O2*W*—H2*W*2···N4^iv^	0.85 (2)	2.05 (2)	2.863 (4)	160 (4)
Mic^+^·Prm5CA^−^ (**5**)				
N2—H2···O3	0.91 (5)	1.73 (5)	2.618 (3)	164 (4)
C3—H3···N4^i^	0.95	2.57	3.249 (3)	128
C5—H5···O2^ii^	0.95	2.21	3.075 (3)	151
C22—H22···O1^ii^	0.95	2.39	3.278 (3)	156
C24—H24···O3^i^	0.95	2.26	3.141 (3)	155

Symmetry codes: (**1**): (i) *x*, *y* − 1, *z*; (ii) *x* + 1, *y*, *z*; (iii) −*x* − 1, −*y* + 1, −*z*. (**2**) (i) −*x*, *y* + 1/2, −*z* + 1/2; (ii) *x*, −*y* + 1/2, *z* + 1/2; (iii) −*x*, −*y* + 1, −*z* + 1; (iv) −*x*, *y* − 1/2, −*z* + 1/2; (v) *x*, *y* − 1, *z*; (vi) −*x* + 1, *y* − 1/2, −*z* + 3/2. (**3**) (i) *x*, *y* − 1, *z*; (ii) −*x* + 1, *y*, −*z* + 1/2; (iii) −*x* + 1, *y* + 1, −*z* + 1/2; (iv) −*x* + 1/2, *y* + 1/2, −*z* + 1/2. (**4**) (i) −*x* + 2, −*y*, −*z* + 1; (ii) *x* − 1, *y*, *z*; (iii) −*x* + 2, −*y*, −*z* + 2; (iv) *x* + 1, *y*, *z*. (**5**) (i) −*x* + 2, −*y* + 1, −*z* + 1; (ii) −*x* + 1, −*y*, −*z* + 1.

**Table 2:** The surface roughness descriptors for miconazole salts (**1**)–(**5**).

Compound	Surface	Rugosity	RMSD	Skewness	Kurtosis
Mic^+^·Pyz2CA^−^·2H_2_O·0.5MeOH (**1**)	(002)	1.922	1.764	0.127	2.241
Mic^+^·Pyz2,3DCA^−^ (**2**)	(200)	2.281	2.426	0.096	2.289
Mic^+^·Pyz2,3DCA^−^ 3H_2_O (**3**)	(002)	1.907	1.825	0.136	2.269
Mic^+^·Pyd3CA^−^ 2H_2_O (**4**)	(020)	1.921	1.782	0.129	2.323
Mic^+^·Prm5CA^−^ (**5**)	(001)	1.374	0.985	0.168	1.601

**Table 3:** The atom properties (counts/A^2^) of the analyzed surfaces for miconazole salts (**1**)–(**5**).

Compound	Surface	Donor Density	Acceptor Density	Aromatic Density
Mic^+^·Pyz2CA^−^·2H_2_O·0.5MeOH (**1**)	(002)	0.133	0.061	0.143
Mic^+^·Pyz2,3DCA^−^ (**2**)	(200)	0.147	0.086	0.178
Mic^+^·Pyz2,3DCA^−^ 3H_2_O (**3**)	(002)	0.126	0.058	0.146
Mic^+^·Pyd3CA^−^ 2H_2_O (**4**)	(020)	0.119	0.079	0.148
Mic^+^·Prm5CA^−^ (**5**)	(001)	0.066	0.053	0.119

**Table 4:** The solubility of miconazole (mg/L or mmol/L) for miconazole salts (**1**)–(**5**).

Compound	Miconazole Concentration in mg/L	Miconazole: Concentration in mmol/L
Mic^+^·Pyz2CA^−^·2H_2_O·0.5MeOH (**1**)	38.22 ± 3.71	0.0323 ± 0.0031
Mic^+^·Pyz2,3DCA^−^ (**2**)	42.45 ± 5.30	0.0727 ± 0.0091
Mic^+^·Pyz2,3DCA^−^·3H_2_O (**3**)	4.09 ± 0.06	0.0064 ± 0.0001
Mic^+^·Pyd3CA^−^·2H_2_O (**4**)	51.39 ± 5.68	0.0892 ± 0.0099
Mic^+^·Prm5CA^−^ (**5**)	43.16 ± 3.31	0.0799 ± 0.0061

**Table 5:** The antifungal properties of miconazole salts (**1**), (**2**), (**4**), and (**5**).

Compound	*C. albicans*		*C. parpasilosis*		*C. glabrata*		*C. auris*	
	MIC	MFC	MIC	MFC	MIC	MFC	MIC	MFC
	Concentration [mg/L]							
(**1**)	4	512	4	512	0.5	nd	4	512
(**2**)	8	nd	4	256	0.5	512	16	512
(**4**)	4	nd	8	512	0.5	512	32	nd
(**5**)	2	512	4	nd	0.5	512	16	nd
Miconazole	32	nd	16	128	0.5	512	32	512

nd—not designated.

## Data Availability

The original contributions presented in this study are included in the article/Supplementary Material. Further inquiries can be directed to the corresponding author.

## Supplementary Materials

The following supporting information can be downloaded at: https://www.mdpi.com/article/10.3390/molecules31101686/s1, Figure S1: Superposition of five miconazole molecules; Figure S2: FT-IR spectra for miconazole salts; Figure S3: FT-IR spectra for pure miconazole and coformers; Table S1: The torsion angle C1—O1—C12—C13 determining a conformation of miconazole cation; Table S2: C—O bond lengths and corresponding differences for carboxylic acids in miconazole salts; Table S3: Geometric parameters of aromatic π–π interactions for miconazole salts; Table S4: Hydrogen-bond donors and acceptors, and their ratio for miconazole salts; Table S5: Crystal data, data collection and structure refinement details for miconazole salts.
