# Supplementary material for: New Miconazole Salts with Heterocyclic Carboxylic Acids with Improved Water Solubility and Enhanced Antifungal Activity
Source: Molecules. 2026 May 16;31(10):1686. doi: 10.3390/molecules31101686 (PMC13209626; doi:10.3390/molecules31101686)
Supplement: Supplementary file 1 [file molecules-31-01686-s001.zip › molecules-4293973-supplementary.pdf]

SUPPORTING INFORMATION

New Miconazole Salts with Heterocyclic Carboxylic Acids with Improved Water Solubility and Enhanced Antifungal Activity

Anna Ben,[1,2,3] Aleksandra Felczak,[4] Michał Gacki,[5] Katarzyna Lisowska,[4] Mateusz R. Gołdyn,[3,6] Elżbieta Bartoszak-Adamska,[3] Lilianna Chęcińska[1*]

[1] Faculty of Chemistry, University of Lodz, Pomorska 163/165, 90-236 Łódź, Poland

[2] University of Lodz Doctoral School of Exact and Natural Sciences, Narutowicza 68, 90-136 Łódź, Poland

[3] Adam Mickiewicz University, Faculty of Chemistry, Uniwersytetu Poznańskiego 8, 61-614 Poznań, Poland

[4] University of Lodz, Faculty of Biology and Environmental Protection, Department of Industrial Microbiology and Biotechnology, Banacha 12/16, 90-237 Łódź, Poland

[5] Lodz University of Technology, Institute of General and Ecological Chemistry, Żeromskiego 114, 90-924 Łódź, Poland

[6] Adam Mickiewicz University, Center for Advanced Technologies, Uniwersytetu Poznańskiego 10, 61-614 Poznań, Poland

* Corresponding Author: lilianna.checinska@chemia.uni.lodz.pl

Table of contents

	Figures and tables	Page
Figure S1	Superposition of five miconazole molecules	2
Table S1	The torsion angle C1–O1–C12–C13 determining a conformation of miconazole cation	2
Table S2	C–O bond lengths and corresponding differences for carboxylic acids in miconazole salts	2
Table S3	Geometric parameters of aromatic π-π interactions for miconazole salts	3
Table S4	Hydrogen-bond donors and acceptors, and their ratio for miconazole salts	3
Figure S2	FT-IR spectra for miconazole salts	4
Figure S3	FT-IR spectra for pure miconazole and coformers	4
Table S5	Crystal data, data collection and structure refinement details for miconazole salts	5

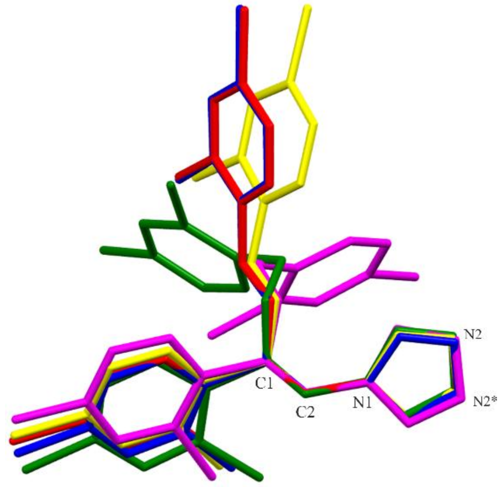

Figure S1. Superposition of five miconazole molecules, showing the best fit for atoms C1, C2, N1; the colour code is red = **Mic**$^+$·Pyz2CA$^-$·2H_2O·0.5MeOH (**1**), green = **Mic**$^+$·Pyz2,3DCA$^-$ (**2**), yellow = **Mic**$^+$·Pyz2,3DCA$^-$ ·3H_2O (**3**), blue = **Mic**$^+$·Pyd3CA$^-$·2H_2O (**4**), and magenta = **Mic**$^+$·Prm5CA$^-$ (**5**).

N2* is a position of the N2 atom in Mic$^+$ molecule in (**2**) and (**5**).

Table S1. The torsion angle C1–O1–C12–C13 (°) determining a conformation of miconazole cation in miconazole salts (**1**) – (**5**)

Structure	Torsion angle C1–O1–C12–C13
Mic$^+$·Pyz2CA$^-$·2H_2O·0.5MeOH (**1**)	174.19(14)
Mic$^+$·Pyz2,3DCA$^-$ (**2**)	69.40(14)
Mic$^+$·Pyz2,3DCA$^-$·3H_2O (**3**)	-176.73(11)
Mic$^+$·Pyd3CA$^-$·2H_2O (**4**)	174.0(2)
Mic$^+$·Prm5CA$^-$ (**5**)	-75.7(2)

Table S2. C–O bond lengths ($d_{C–O}$ in Å) and corresponding differences ($\Delta D_{C–O}$ in Å) for carboxylic acids in miconazole salts (**1**) – (**5**)

Structure	Group	$d_{C–O}$	$d_{C–O}$	$\Delta D_{C–O}$
Mic$^+$·Pyz2CA$^-$·2H_2O·0.5MeOH (**1**)	COO$^-$	1.258(3)	1.249(3)	0.009(4)
Mic$^+$·Pyz2,3DCA$^-$ (**2**)	COO$^-$	1.2421(16)	1.2740(16)	0.0319(23)
	COOH	1.2112(16)	1.3154(16)	0.1042(23)
Mic$^+$·Pyz2,3DCA$^-$·3H_2O (**3**)	COO$^-$	1.2735(18)	1.2331(19)	0.0404(26)
	COOH	1.2164(19)	1.295(2)	0.0786(28)
Mic$^+$·Pyd3CA$^-$·2H_2O (**4**)	COO$^-$	1.253(3)	1.248(3)	0.005(4)
Mic$^+$·Prm5CA$^-$ (**5**)	COO$^-$	1.240(3)	1.269(3)	0.029(4)

Table S3. Geometric parameters (Å, °) of aromatic π-π interactions for miconazole salts (**1**) – (**5**)

1 – imidazole ring; 2 – phenyl ring C6-C11; 3 – phenyl ring C13-C18; 4 – heterocyclic ring in acids

Structure	Contact	*Cg*I···*Cg*J	α	CgI_{perp}	CgJ_{perp}	Slippage
$Mic^{+}\cdot Pyz2CA^{-}\cdot 2H_2O\cdot 0.5MeOH$ (**1**)	*Cg*1···*Cg*4^{i}	3.6817(12)	2.49(11)	3.2872(8)	3.3534(8)	1.520
	*Cg*2···*Cg*4^{ii}	3.9947(12)	14.17(10)	3.1628(9)	3.5801(9)	1.772
	*Cg*3···*Cg*3^{iii}	3.8035(11)	0.02(9)	3.3470(8)	3.3471(8)	1.807
$Mic^{+}\cdot Pyz2{,}3DCA^{-}$ (**2**)	*Cg*3···*Cg*3^{ii}	3.8467(8)	0.02(7)	3.4749(6)	3.4749(6)	1.650
	*Cg*4···*Cg*2^{iii}	3.7889(8)	12.16(6)	3.4376(5)	3.6959(6)	0.834
$Mic^{+}\cdot Pyz2{,}3DCA^{-}\cdot 3H_2O$ (**3**)	*Cg*3···*Cg*3^{i}	3.9195(8)	0.03(7)	3.5606(6)	3.5605(6)	1.638
$Mic^{+}\cdot Pyd3CA^{-}\cdot 2H_2O$ (**4**)	*Cg*3···*Cg*3^{i}	3.8600(15)	0.03(12)	3.3865(10)	3.3865(10)	1.852
$Mic^{+}\cdot Prm5CA^{-}$ (**5**)	*Cg*1···*Cg*4^{i}	3.8343(14)	21.98(13)	2.9959(10)	3.6575(10)	
	*Cg*3···*Cg*3^{iii}	3.7288(16)	0.04(13)	3.5212(11)	3.5212(11)	1.227

Symmetry codes: $Mic^{+}\cdot Pyz2CA^{-}\cdot 2H_2O\cdot 0.5MeOH$ (i) –*x*, –*y*+1, –*z*; (ii) *x*, *y*–1, *z*; (iii) –*x*+2, –*y*+1, –*z*+1; $Mic^{+}\cdot Pyz2{,}3DCA^{-}$ (ii) –*x*+1, –*y*, –*z*+1; (iii) *x*, –*y*+1/2, *z*–1/2; $Mic^{+}\cdot Pyz2{,}3DCA^{-}\cdot 3H_2O$ (i) –*x*+1, –*y*–1, –*z*+1; $Mic^{+}\cdot Pyd3CA^{-}\cdot 2H_2O$ (i) –*x*, –*y*+1, –*z*+1; $Mic^{+}\cdot Prm5CA^{-}$ (i) *x*–1, *y*, *z*; (iii) –*x*+1, –*y*, –*z*+2.

Cg(I)···*Cg*(J) – distance between ring centroids; α - dihedral angle between planes I and J; $Cg(I)_{perp}$ and $Cg(J)_{perp}$ - (interplanar spacing) perpendicular distance of *Cg*(I) on ring J and *Cg*(J) on ring I, respectively; slippage - distance between *Cg*(I) and perpendicular projection of *Cg*(J) on ring I.

Table S4. Hydrogen-bond donors and acceptors, and their ratio for miconazole salts (**1**) – (**5**)

	H-bond donors (D)* (heteroatoms)	H-bond acceptors (A)	D:A ratio	Unsatisfied H-bond acceptors[#]
$Mic^{+}\cdot Pyz2CA^{-}\cdot 2H_2O\cdot 0.5MeOH$ (**1**)	1N (imidazole)<br>4O (water)<br>1O (methanol)	2N<br>2O (carboxylic)<br>2O (water)<br>1O (methanol)	6:7	–
$Mic^{+}\cdot Pyz2{,}3DCA^{-}$ (**2**)	1N (imidazole)<br>1O (carboxylic)	2N<br>3O (carboxylic)	2:5	1N
$Mic^{+}\cdot Pyz2{,}3DCA^{-}\cdot 3H_2O$ (**3**)	1N (imidazole)<br>6O (water)<br>1O (carboxylic)	2N<br>3O (carboxylic)<br>3O (water)	8:8	–
$Mic^{+}\cdot Pyd3CA^{-}\cdot 2H_2O$ (**4**)	1N (imidazole)<br>4O (water)	2N<br>2O (carboxylic)<br>2O (water)	5:6	–
$Mic^{+}\cdot Prm5CA^{-}$ (**5**)	1N (imidazole)	2N<br>2O (carboxylic)	1:4	1N

* (C)–H donor groups were excluded, only heteroatoms were counted; additionally, the four chlorine atoms were excluded from hydrogen-bonding acceptors as typically they do not participate in hydrogen bonds in miconazole structures

[#] Unsatisfied H-bond acceptors were counted after hydrogen-bonding analysis including C–H···O/N interactions.

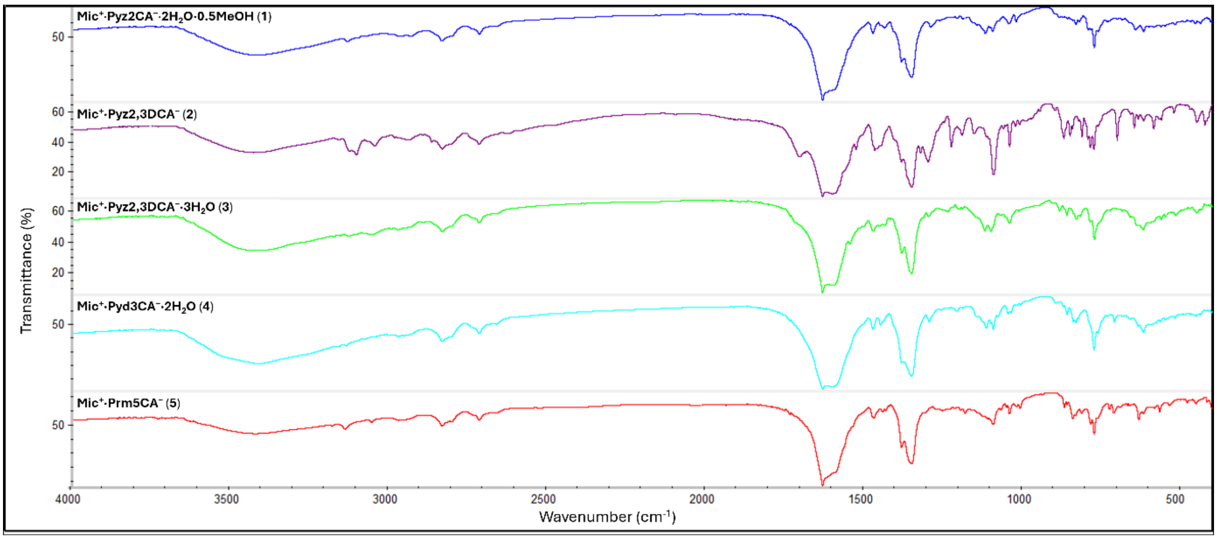

Figure S2. FT-IR spectra for miconazole salts: Mic^{+}·$Pyz2CA^{-}$·$2H_2O$·0.5MeOH (**1**), Mic^{+}·$Pyz2,3DCA^{-}$ (**2**), Mic^{+}·$Pyz2,3DCA^{-}$·$3H_2O$ (**3**), Mic^{+}·$Pyd3CA^{-}$·$2H_2O$ (**4**), Mic^{+}·$Prm5CA^{-}$ (**5**).

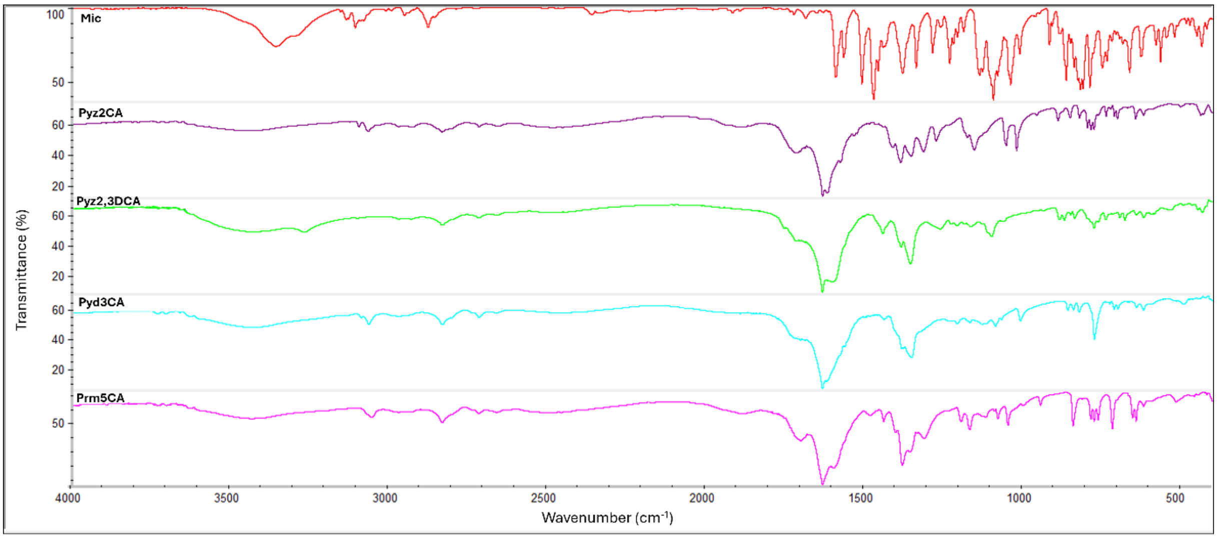

Figure S3. FT-IR spectra for pure miconazole and coformers: Pyz2CA, Pyz2,3DCA, Pyd3CA, Prm5CA.

Table S5. Crystal data, data collection and structure refinement details for miconazole salts (**1**) – (**5**) (experiments were carried out at 100K with Cu *K*α radiation)

	$Mic^{+}\cdot Pyz2CA^{-}\cdot 2H_2O\cdot 0.5MeOH$ (**1**)	$Mic^{+}\cdot Pyz2,3DCA^{-}$ (**2**)	$Mic^{+}\cdot Pyz2,3DCA^{-}\cdot H_2O$ (**3**)	$Mic^{+}\cdot Pyd3CA^{-}\cdot 2H_2O$ (**4**)	$Mic^{+}\cdot Prm5CA^{-}$ (**5**)
CCDC number	2522078	2522079	2522080	2522081	2522082
Crystal data					
Chemical formula	$2(C_{18}H_{15}Cl_4N_2O)\cdot 2(C_5H_3N_2O_2)\cdot 4(H_2O)\cdot CH_3OH$	$C_{18}H_{15}Cl_4N_2O\cdot C_6H_3N_2O_4$	$C_{18}H_{15}Cl_4N_2O\cdot C_6H_3N_2O_4\cdot 3(H_2O)$	$C_{18}H_{15}Cl_4N_2O\cdot C_5H_3N_2O_2\cdot 2(H_2O)$	$C_{18}H_{15}Cl_4N_2O\cdot C_5H_3N_2O_2$
M_r	1184.53	584.22	638.27	576.24	540.21
Crystal system, space group (Z)	Triclinic, $P\bar{1}$ (Z=1)	Monoclinic, $P2_1/c$ (Z=4)	Monoclinic, $C2/c$ (Z=8)	Triclinic, $P\bar{1}$ (Z=2)	Triclinic, $P\bar{1}$ (Z=2)
a, b, c (Å)	8.0560 (2), 12.4575 (3), 13.4940 (3)	15.0239 (1), 11.2852 (1), 14.4304 (1)	25.8049 (4), 7.9664 (1), 28.1278 (4)	8.1485 (1), 12.3256 (3), 12.7127 (2)	8.4747 (2), 8.9484 (3), 15.3128 (5)
α, β, γ (°)	99.535 (1), 101.902 (1), 102.819 (1)	90, 94.141 (1), 90	90, 110.143 (2), 90	84.538 (2), 77.624 (1), 84.508 (2)	90.364 (3), 94.076 (2), 91.372 (2)
V ($Å^3$)	1259.97 (5)	2440.25 (3)	5428.62 (15)	1237.74 (4)	1157.94 (6)
μ (mm^{-1})	4.68	4.81	4.46	4.73	4.95
Crystal size (mm)	0.42 × 0.09 × 0.09	0.18 × 0.14 × 0.03	0.26 × 0.09 × 0.04	0.24 × 0.05 × 0.03	0.41 × 0.07 × 0.02
Data collection					
Diffractometer/detector	Bruker D8 Quest/ Photon-III	Rigaku XtaLAB Synergy, Dualflex/HyPix	Rigaku XtaLAB Synergy, Dualflex/Pilatus 300K	Rigaku XtaLAB Synergy, Dualflex/HyPix	Rigaku XtaLAB Synergy, Dualflex/HyPix
Absorption correction	Multi-scan	Gaussian	Gaussian	Gaussian	Gaussian
T_{min}, T_{max}	0.252, 0.688	0.271, 1.000	0.758, 1.000	0.322, 0.974	0.178, 1.000
No. of measured, independent and observed [$I > 2\sigma(I)$] reflections	45797, 4612, 4481	23877, 4916, 4766	31923, 5678, 5164	23067, 4756, 4509	12339, 4407, 4116
R_{int}	0.032	0.025	0.033	0.030	0.033
$(\sin \theta/\lambda)_{max}$ ($Å^{-1}$)	0.603	0.632	0.636	0.617	0.617
Refinement					
$R[F^2 > 2\sigma(F^2)]$, $wR(F^2)$, S	0.034, 0.092, 1.05	0.027, 0.069, 1.03	0.028, 0.072, 1.05	0.039, 0.100, 1.19	0.044, 0.127, 1.09
No. of reflections /parameters/restraints	4612/356/6	4916/342/0	5678/393/1	4756/345/4	4407/311/0
$\Delta\rho_{max}$, $\Delta\rho_{min}$ (e $Å^{-3}$)	0.56, -0.45	0.45, -0.27	0.32, -0.25	0.43, -0.28	0.53, -0.40
